# Increasing the deterrence of airport security checks by managing expectations through communication: a hypothetical scenario experiment

**DOI:** 10.1007/s12198-021-00240-8

**Published:** 2021-11-25

**Authors:** Angela Bearth, Franziska Hofer, Tamara Stotz, Signe Ghelfi

**Affiliations:** 1 Consumer Behavior, Institute for Environmental Decisions (IED), ETH Zurich, CHN J 75.2, Universitaetstrasse 22, 8092 Zurich, Switzerland; 2 Airport Division – Research and Development, Zurich State Police, P.O. Box 8058, Zurich, Switzerland; 3 HF Partners GmbH, Sonneggstrasse 86, 8006 Zürich, Switzerland

**Keywords:** Airport security, Deterrence, Decision making, Unpredictability, Perception

## Abstract

Selective security screenings are discussed as a potential strategy to reduce costs and waiting times at airports, while keeping security high. However, the limited literature suggests that traditional security screenings, where all passengers are screened, are perceived as more deterrent for criminal activity and more secure from passengers’ perspectives. The goal of this study was to investigate whether targeted communication on an airport’s website can counteract the detrimental effect of randomised airport security checks on deterrence. The study results confirm prior findings that people with illegal intentions prefer randomised security checks compared to traditional security checks. However, there are hints that tactical communication could be a tool to improve security at airports. All in all, the insights gathered in this study should be taken as a sign of caution, when considering switching to selective security screenings. Future directions for investigating the effect of tactical communication are discussed.

## Introduction and theoretical background

US late night show host, Trevor Noah, joked about the Transportation Security Administration’s plans to reduce security screening at smaller airports in the US, which sounded like a crazy and dangerous idea (Noah 2018; Transportation Security Administration (TSA) 2020). However, selective security screenings have been discussed in the literature as a potential strategy to reduce costs and waiting times at airports, while simultaneously keeping security high (Haldimann 2017; Nguyen & John 2018; Scurich & John 2014). This originates from the premise that making security checks less predictable would reduce the ability of insiders and observers to identify and take advantage of security gaps (Haldimann 2017; Loffi & Wallace 2014; Wallace & Loffi 2014). Selective screening requires a strategy to select passengers for screening, either by introducing randomness, screening for suspicious behaviour or by applying risk-based approaches (Koller et al. 2016; Mann et al. 2020; Vrij et al. 2020).

In policing and airport security, unpredictability is currently discussed as a key strategy to reduce a variety of threats at airports. Many airports have already implemented different measures to make security processes less predictable. For example, at Los Angeles Airport, a software is implemented that generates randomness in security checkpoints and patrol routes (i.e., assistant for randomized monitoring over routes ARMOR) (Pita et al. 2009). Furthermore, the government of the United Kingdom deployed a project with a strong focus on uncertainty and communication of security measures in order to deter, detect and disrupt criminal activity (i.e., Project Servator) (Hicks 2018). The focus lies on the combination of influence activity (e.g., through highly visible police interventions and/or active communication via social media) and behaviour detection (i.e., reaction to interventions, such as security check points) (Hicks 2016). This includes the communication of specific activities and security measures through various channels such as social media, posters, and media. The program seeks to be unpredictable to increase deterrence.

The usefulness of randomness in airport security is backed theoretically by people’s use of heuristics when making judgments or taking decisions under uncertainty (e.g., when knowledge is lacking regarding choices and outcomes). Heuristics are simple rules-of-thumb that people use to reduce cognitive load in judgment and decision making (Gigerenzer & Gaissmaier 2011; Keller et al. 2006; Slovic et al. 2007). This means that people tend to base their decisions on certain cues instead of weighing all the information at hand as predicted by economic theory (e.g., game theory, see Osborne & Rubinstein 1994). Consequently, the resulting decision might deviate from what could be expected from a purely rational perspective (e.g., subjective expected utility). For example, it is known from economic and psychological research that people prefer risk (e.g., with known likelihoods of occurrence) compared to uncertainty or ambiguity when making decisions (Ellsberg 1961; Fox & Weber 2002; Machina & Siniscalchi 2014).

Particularly, the affect heuristic has been used to describe human judgment and decision making in various situations (Finucane et al. 2000; Slovic et al. 2007). According to the affect heuristic, people tend to make decisions based on the positive or negative affect raised from a particular cue in the decision situation. Cues can refer to aspects of a particular situation (e.g., visible aspects, signs), but also to other types of information (e.g., memories, prior experiences). Research on behavioural change suggests that cues are considered to be relatively effective intervention types (Osbaldiston & Schott 2012). Thus, specific information and cues about randomised, unpredictable security checks at airports could lead to a deterrence effect for people with a criminal intent. The underlying mechanism could lie in the affect heuristic, i.e. cues about randomness could lead to an affective impression of uncertainty in the way that it is impossible to predict the risk of being uncovered (Ghelfi et al. 2019; Haldimann 2017). However, these mechanisms have been primarily studied in experiments involving gambling tasks and economic decision making or for other decisional contexts, such as environmental, medical or financial decision making (Fox & Weber 2002; Gigerenzer & Gaissmaier 2011). Hence, a solid foundation of scientific evidence of the mechanisms at work when people judge airport security are currently missing.

Researchers interested in the effect of increased uncertainty at airports focused on the criminal perspective and on the perspective of passengers so far. The findings of these studies suggest partly detrimental effects of changing airport security practices from the status quo to alternative security checks, namely on deterrence of criminal activities and on fairness and security perception at airports (Nguyen & John 2018; Nguyen et al. 2017; Scurich & John 2014; Stotz et al. 2020, 2021). Particularly, randomised security checks (i.e., where only a random sample of passengers are screened) were associated with a higher perceived likelihood of being able to successfully smuggle an explosive dummy when compared to traditional security checks^1^ (i.e., where all passengers are screened) (Stotz et al. 2020). This effect was uncovered regardless of whether the majority (i.e., 90 %) or only a minority of passenger (i.e., 30 %) are screened (Stotz et al. 2020). This suggests that the participants did not consider the likelihood of being checked (30-90 %) to determine their potential success as smugglers, but rather fundamentally differentiated between the traditional security check and an alternative security measure, which allows some passengers to pass through without being checked. Similar findings regarding the reduction of deterrent effect of randomised security checks were obtained by Stotz et al. (2021), although it was explicitly stated that both kinds of security checks did not differ in detection rate. Scurich and John (2014) investigated passengers’ judgment of traditional (all passengers are searched, 1 in x contraband detected) and randomised security checks (1 in x passengers are searched, of the searched passengers all contraband will be detected). The authors found that randomised security checks were seen as more convenient, whereas traditional security checks were seen as safer and fairer (Scurich & John 2014). Similar findings were obtained by authors investigating the impact of selective screening on security and fairness perception of airport passengers (Nguyen & John 2018; Nguyen et al. 2017).

Overall, the current state of research suggests that changing the status quo of traditional security checks at airports (a) might lead to lower deterrence in criminals and (b) might be perceived as unfair, which in turn might reduce acceptability of security checks and thus, cooperation of passengers. However, the above-mentioned studies were based on rather abstract scenario experiments, where participants were presented with descriptions of different types of security checks. It is unclear, whether the findings would translate to a more realistic scenario. Thus, the aim of the study was twofold: First, we investigated whether additional communication measures, signalling unpredictability of security measures, can counteract the detrimental effect of randomised airport security checks. Second, the study aimed at adding the criminal perspective on traditional vs. randomised airport security checks to the current state of research. Lastly, it aimed at investigating the relationships between deterrence, perceived likelihood of success, certainty, and personality variables.

## Study goals and research questions

The goal of this study was to broaden the knowledge about people’s perceptions of security checks at airports from a criminal’s perspective (i.e., smuggling illegal objects through the security check). For this, the type of security check (traditional vs. randomised) and the level of communicated unpredictability (i.e., unpredictability cue) were varied on the website of a fictional airport. Specifically, the following research questions (RQ) were investigated:

- RQ 1: Does the type of security check implemented at the airport (traditional vs. randomised) and the presence of an unpredictability cue impact the choice of day to smuggle the illegal objects and the perceived certainty related to this choice?
- RQ2: What reasons do participants indicate for choosing a particular day to smuggle the illegal objects (weekdays vs. weekends)?
- RQ3: Does the type of security check implemented at the airport (traditional vs. randomised) and the presence of an unpredictability cue impact the perceived likelihood of success and deterrence?
- RQ4: Are an individual’s willingness to breach social norms, and sociodemographic variables related to their estimated certainty, likelihood of success and deterrence?

## Methodology

### Study design and material

This was a hypothetical scenario experiment with a 2 (type of security check: randomised versus traditional) by 2 (unpredictability cue: present versus not present) design, loosely based on prior scenario experiments (Scurich and John 2014; Stotz et al. 2020, 2021). The participants were introduced to the scenario with the following text:

‘On the following page, we will present you with a fictional scenario. Please read the scenario carefully and try to empathise with it as much as possible. It might be best to think of it as a game, with chances of success but also risks and consequences. It is important for us that you consider these carefully and make the most successful decision possible. Imagine that you are in a financial emergency. A distant acquaintance offers you a large sum of money if you help him smuggle a small parcel with illegal items abroad by plane. You must make it to the departure gate at the airport without the parcel, containing the illegal items, being discovered. Due to your financial situation, you decide to accept the offer.’

**Fig. 1:**
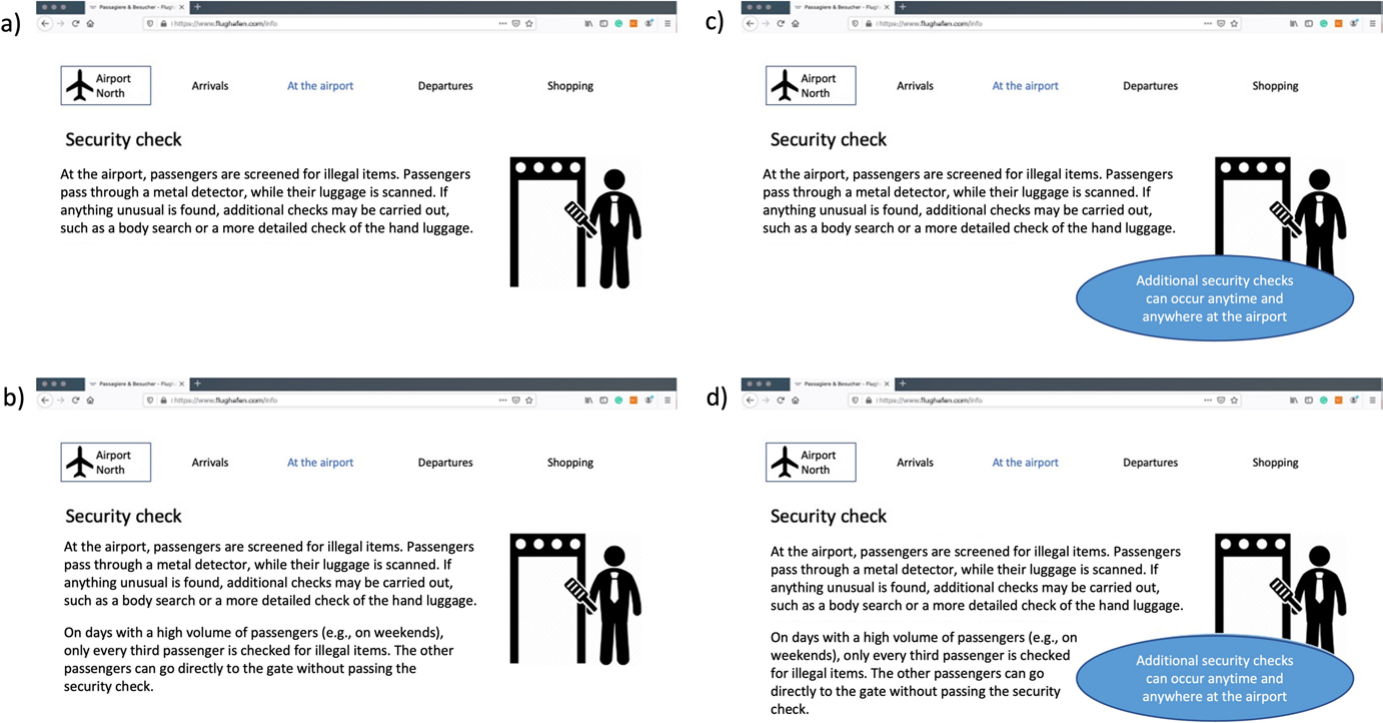
The four websites that were presented to the participants: (**a**) traditional security check, (**b**) randomised security check, (**c**) traditional security check with unpredictability cue, (**d**) randomised security check with unpredictability cue

After this introductory text, the participants were randomly split into four groups based on the previously presented 2 × 2 design. Next, the participants were informed that prior to booking a flight, they first had the opportunity to gather information about the security check from the airport’s website. The four groups were then presented with pictures of four different websites, which were modelled after existing airport websites. The airport logo on this website, as well as the URL, were fictitious. Figure 1 shows the pictures of the four websites presented to the participants. All websites featured a description of the regular security check. In the two groups in the randomised condition (b and d in Fig. 1), two sentences were added on the security measures during times with high passenger volumes. In the two groups in the ‘unpredictability cue present’ condition (c and d in Fig. 1), a light-blue box was added that reminded participants of the additional security checks that could occur anytime and anywhere at the airport.

After the experimental manipulation, the participants were asked whether they would book the flight on a weekday (Monday to Friday) or on a weekend (Saturday or Sunday), as well as for the reason for their choice in an open response field. Additionally, they were asked how certain they were that they chose the right day to attempt to smuggle the parcel successfully on a slider from 1 ‘not sure at all’ to 11 ‘very sure.’ The likelihood of success was measured with the item ‘How high do you estimate the likelihood that you can successfully smuggle this illegal package without being detected?’ on a slider from 1 ‘very low’ to 11 ‘very high.’ Deterrence was measured with the item ‘How deterred are you by these security measures?’ on a slider from 1 ‘not deterred at all’ to 11 ‘very deterred.’

The participants who filled the questionnaire out on a desktop computer were then forwarded to a second section of the questionnaire for another study (i.e., Implicit Association Test), while the participants filling the questionnaire out on a tablet or mobile were directly led to the last part and end of the questionnaire. The results of this other study were not part of this study and will be presented elsewhere. In this last part, three items were included to measure participants’ willingness to breach social norms on a scale from 1 ‘do not agree at all’ to 5 ‘fully agree.’ The three items resulted in an internal consistency of α = 0.55. Table 1 presents the descriptives of the three items and scale. Participants were also asked about their sex, age, educational level and familiarity with airports (‘How often do you spend time at airports? Please think of the time before the coronavirus crisis’ with response options 1 ‘(almost) every day,’ 2 ‘a few times per week,’ 3 ‘a few times per month,’ 4 ‘a few times per year’ and 5 ‘less frequently or never’). Lastly, participants were asked whether they had any remarks about the study in an open response field.

**Table 1:** Means (M), standard deviations (SD) and corrected item-total correlations (r_i_) of willingness to breach social norms (1 ‘do not agree at all’ to 5 ‘fully agree;’ N = 510)

Willingness to breach social norms	M	SD	r_i_
If I do not feel like doing something (e.g., working, going to a party), I just call in sick.	1.71	0.99	0.37
If I get too much change while paying the bill in a restaurant, I keep it to myself.	2.05	1.18	0.33
It is acceptable to not declare everything you have with you at border customs (e.g., more meat or wine than allowed).	2.25	1.22	0.40

### Study sample

Recruiting was supported by a professional market research company (respondi). The participants were invited via link to participate in this study and received a monetary incentive after participating. Quota sampling based on age and sex was applied to ensure a heterogenous sample of participants. Prior to data analysis, the data was cleaned by inspecting the time that participants took to fill out the questionnaire (speeders: less than 1 min) and the responses that they provided in the open response fields (e.g., ‘I did not see a reason for filling out this questionnaire’). Based on this, *n* = 48 participants were excluded. Thus, a sample of *N* = 510 participants was reached (*n* = 260 female participants (51 %), *M*_age_ = 45, *SD*_*age*_ = 15). The sample comprised *n* = 208 participants with a high educational level (41 %; higher educational institution or university) and *n* = 302 participants with a lower educational level (59 %). The participants’ familiarity with airports varied with the majority of participants being at the airport a few times per year (*n* = 237, 47 %) or less frequently (*n* = 236, 45 %). A total of *n* = 24 participants (5 %) were at airports a few times per month, *n* = 9 (2 %) a few times per week and *n* = 4 (1 %) (almost) every day.

### Data Analysis

All descriptive and multivariate analyses were done in SPSS 26.0 (IBM Corp. 2017). The multi-item scales were subjected to a scale analysis for internal consistency (Cronbach’s alpha) prior to data analysis. The open responses regarding the reason for choosing either a weekday or weekend were categorised by two separate coders (intercoder reliability: Cohen’s Kappa = 0.75). If participants mentioned several associations in the open response field, only the first association was coded. Differences in coding by the two coders were resolved by the corresponding author. For the experimental effect, a three-way loglinear analysis with follow-up Chi^2^-tests (likelihood ration: χ^2^(4) = 3.31, *p* = .507) and separate univariate ANOVAs were conducted. Lastly, bivariate correlations and an independent t-test were conducted to test for relationships among the variables and sociodemographics.

## Results

### Choice of day to smuggle the illegal objects, certainty and reasons for the choice

**Fig. 2:**
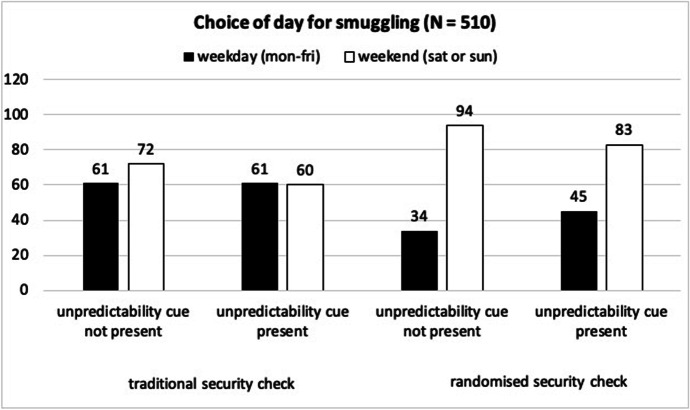
Distribution of responses regarding the day that participants would attempt to smuggle, separated by experimental conditions (N = 510)

Figure 2 presents the distribution of responses regarding the choice of day for smuggling. The participants in both randomised conditions were significantly more likely to pick the weekend (i.e., higher passenger volume and randomized checks) than participants in both traditional conditions (choice x type of security check: χ^2^(1) = 15.75, *p* < .001). The presence of an unpredictability cue had no significant impact on the participants’ choice (choice x unpredictability cue: χ^2^(1) = 2.03, *p* = .154) and, there was no three-way interaction effect (choice x type of security check x unpredictability cue: χ^2^(1) = 0.36, *p* = .549). Table 2 presents the descriptive results regarding the participants’ certainty to have chosen the right day to smuggle the parcel successfully. The participants in the randomised condition were significantly more certain about their choice of day to smuggle than the participants in the traditional condition, *F*(1, 506) = 26.33, *p* < .001, η^2^ = 0.05, while the unpredictability cue did not have a significant impact on certainty, *F*(1, 506) = 0.02, *p* = .883, η^2^ = 0.00, and there was no significant interaction effect, *F*(1, 506) = 0.71, *p* = .400, η^2^ = 0.00.

**Table 2:** Descriptives for certainty regarding choice of day (1 ‘very uncertain’ to 11 ‘very certain’), likelihood of success (1 ‘very low’ to 11 ‘very high’) and deterrence (1 ‘not deterred at all’ to 11 ‘very deterred’)

	Type of security check traditional		Type of security check randomised	
	Unpredictability cue not present	Unpredictability cue present	Unpredictability cue not present	Unpredictability cue present
	M (SD)	M (SD)	M (SD)	M (SD)
Certainty regarding choice of day	6.58 (2.58)	6.80 (2.65)	7.92 (2.41)	7.77 (2.51)
Likelihood of success	4.89 (2.37)	4.78 (2.55)	6.03 (1.94)	5.97 (2.09)
Deterrence	8.15 (2.29)	7.78 (2.46)	7.67 (2.40)	8.09 (2.08)

Of the participants that chose to smuggle on weekdays, the most frequently mentioned reason was a) that less people would travel and thus, less security personnel would be present (*n* = 68). Other reasons that were given for smuggling on a weekday were that more people would be present (*n* = 28), it would be cheaper (*n* = 25), there would be more business travellers at the airport, which would allow to blend in (*n* = 21). Of the participants that chose to smuggle on a weekend, the most frequently mentioned reason was that more people would be present at the airport, which might overwhelm the security personnel (*n* = 165). Other reasons that were given were that more tourists or families would be travelling that distract the security personnel (*n* = 14). The participants in the experimental group (type of security check: randomised) frequently mentioned the randomised security checks on weekends as the reasons for smuggling on a weekend (*n* = 99) or on a weekday (*n* = 6).

### Perceived likelihood of success and deterrence

Table 2 presents the descriptive results regarding likelihood of success and deterrence. Participants in the randomised condition perceived the likelihood of successfully smuggling the illegal objects as higher than the participants in the traditional condition, *F*(1, 506) = 34.42, *p* < .001, η^2^ = 0.06. The presence of an unpredictability cue did not have an impact on the perceived likelihood of success, *F*(1, 506) = 0.19, *p* = .664, η^2^ = 0.00, and there was no significant interaction effect, *F*(1, 506) = 0.01, *p* = .904, η^2^ = 0.00.

**Fig. 3:**
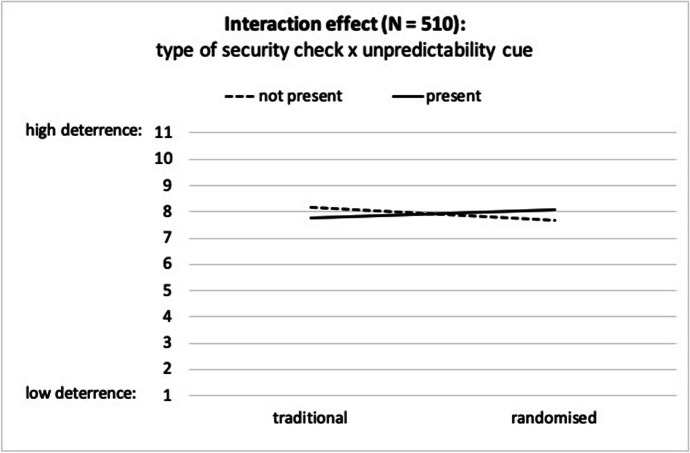
Interaction effect of type of security check and unpredictability cue (*p* = .055; N = 510)

For deterrence, there was solely a marginally significant interaction effect, *F*(1, 506) = 3.70, *p* = .055, η^2^ = 0.01. The main effects of type of security check, *F*(1, 506) = 0.17, *p* = .679, η^2^ = 0.00, and unpredictability cue, *F*(1, 506) = 0.01, *p* = .921, η^2^ = 0.00, were not significant. Figure 3 presents the interaction effect. The unpredictability cue had different impacts on deterrence for the traditional and for the randomised condition: While the unpredictability cue decreased deterrence in the traditional condition it increased deterrence in the randomised condition.

### Relationship among the variables and sociodemographics

Table 3 shows the bivariate correlations between the participants’ individual willingness to breach social norms, their age and the variables of interest (i.e., certainty regarding choice of day, likelihood of success and deterrence). Participants that were more certain to have picked the correct day to smuggle the illegal objects also perceived the likelihood of success to be higher and were less deterred by the security measures. A higher perceived likelihood of success was also associated with lower deterrence. Participants that indicated a higher willingness to breach social norms indicated a higher perceived likelihood of success and lower deterrence. Age was related only to deterrence and willingness to breach social norms: Older participants were more deterred and less willing to breach social norms.

**Table 3:** Bivariate correlations among certainty, likelihood of success, deterrence, willingness to breach social norms and age (Pearson, N = 510)

	Certainty	Likelihood of success	Deterrence	Willingness to breach social norms	Age
Certainty regarding choice of day	-				
Likelihood of success	0.43*	-			
Deterrence	-0.16*	-0.22*	-		
Willingness to breach social norms	0.06	0.17*	-0.11*	-	
Age	-0.00	-0.01	0.09*	-0.32*	-

*: *p* < .05

Sex was not significantly associated with perceived likelihood of success (*t*(508) = -1.23, *p* = .220, Cohen’s *d* = 0.11) and deterrence (*t*(508) = 0.90, *p* = .371, Cohen’s *d* = 0.08). However, men (*M* = 7.56, *SD* = 2.39) felt significantly more confident in their choice of day compared to women (*M* = 6.98, *SD* = 2.76; *t*(508) = -2.55, *p* = .011, Cohen’s *d* = 0.22). Additionally, men (*M* = 2.13, *SD* = 0.84) expressed a higher willingness to breach social norms than women (*M* = 1.89, *SD* = 0.78; *t*(508) = -3.31, *p* = .001, Cohen’s *d* = 0.29). Education was not related to any of the variables of interest (*t*(508) < 0.16, *p* > .877).

## Discussion and directions for the future

Our study adds the following key findings to the existing research on the perception of randomised or risk-based security checks at airports (Nguyen and John 2018; Nguyen et al. 2017; Scurich and John 2014; Stotz et al. 2020, 2021).

First, our study confirms prior findings (Scurich and John 2014; Stotz et al. 2020, 2021) that people with illegal intentions might prefer randomised security checks compared to traditional security checks (i.e., communicated through the airport’s website). This can be seen in the results that participants in the randomised condition were more likely to smuggle on weekends when it was implied that randomised checks would happen more often, were more certain about this choice and expressed higher perceived likelihood of successfully smuggling the illegal objects. In addition, participants were more certain about their choice of day to smuggle the illegal object, when they were confronted with randomised security checks compared to traditional security checks. Both of these findings should be taken as a sign of caution, when considering switching to randomised (or even risk-based) security screenings. Moreover, the results suggest that the addition of an unpredictability cue – in this case a reminder that additional security checks can occur anytime and anywhere at the airport – might increase deterrence for randomised security checks. Oppositely, the unpredictability cue had a negative impact on the deterrence of traditional security checks, as perhaps it led participants to assume that the regular security check was not done with the necessary care and thus, additional security checks might be needed. However, it should be noted that this interaction effect was found only for deterrence and not for likelihood of success. Moreover, the effect size of the interaction effect of type of security check and unpredictability cue is very small. Future studies should attempt to replicate this finding with a larger sample or perhaps a stronger unpredictability cue. This could be achieved by making it more explicit which entity is conducting the unpredictable checks (e.g., the police) and by amplifying the affective response to the cue (e.g., including a picture). In any case, this could be taken as a sign that unpredictability cues can increase deterrence for randomised security checks. It also highlights that it is important to evaluate tactical communication about security measures before implementation to avoid unexpected and backfiring effects (e.g., reduced deterrence, passengers feeling uncomfortable at the airport) (Lirn and Sheu 2009; Nordfjærn and Rundmo 2018; Savage 2012).

Second, deterrence was generally high, even for randomised security checks (above 7 on a scale from 1 to 11). Nonetheless, randomised security checks were seen as slightly easier to breach than traditional security checks, as shown by the values on likelihood of successfully smuggling the illegal object. The open responses show that the key aspects that people are considering are their expectations regarding the situation at the airport and the effectiveness of the current, compared to the new security measure. For both types of security check, the participants offered some strategies to increase their likelihood of success, such as picking a busy day, where security personnel might be overwhelmed, or by blending in with the other travellers. Generally, the numbers and behaviours of fellow passengers are seen as potentially beneficial for illegal activities. Interestingly, willingness to breach social norms was positively related to people’s estimated likelihood of success and negatively related to deterrence. This stresses that in future research into people’s perceptions of airport security, it might be beneficial to consider individual or personality variables too. To sum it up, what might deter some people, might not deter other people based on their personality or individual attitudes.

For future research, it might be beneficial to focus on explaining the processes that occur when people think about security measures at airports and how these might lead to the estimation that security checks at airport are weak and thus, could be identified as a target by criminals. Tactical communication on various channels (e.g., airport websites, at airports) has the potential to change people’s judgments and decisions by changing their expectations regarding airport security. An interesting attribute of tactical communication is that this effect on people’s judgment and decision making can occur without having to change any security measures at all. Thus, tactical communication could potentially be a cost-effective tool to increase the deterrent effect of security measures at airports. From medical science it is known that the mere belief in a treatment effect can have a large influence on the effects that patients feel after starting a new medical treatment (Price et al. 2008). Such placebo effects can be achieved by providing specific information or by more subtle processes, such as previously made experiences or the provision of cues. The term Nocebo describes the negative effects generated by the expectations about a medication’s negative side effects (Faasse and Petrie 2013; Prediger et al. 2019). Transferring these findings on airport security, we argue that actively communicating about additional security measures at airports might influence the expectations of criminals about being detected. Providing targeted information about security measures could therefore strategically be used to increase deterrence without changing any actual measures in place. Future studies could utilise the existing insights from medical sciences and transfer these to the topic of airport security measures to uncover positive and negative effects of communication on airport security.

### Limitations

Overall, the investigation of the effectiveness of deterrent communication strategies at airports is a gold standard and thus, should be the focus of research. However, investigations in real-life settings are difficult to realise for various reasons (e.g., security threat, disruption of security measures in place, generation of loopholes). By conducting an online experiment, a relevant contribution to the state of research in this area can be provided. It is nonetheless important to mention a number of limitations related to this study as they have implications for the generalisability of the study’s findings. First, despite the realistic setting, the results of this study are based on a hypothetical scenario experiment with non-criminal participants. Based on the open responses, it was clear that some people had difficulties to imagine a scenario where they would smuggle something at an airport and furthermore, some participants found the thought of this offensive. Thus, it is possible that the deterrence effect is overestimated in our study. Second, this study was conducted in Switzerland, where no major terrorist attacks occurred at the airport since the 1970 s and thus, trust in airport security is high (Staubli 2017; Szvircsey Tresch et al. 2020). Thus, it might be difficult to transfer our findings to other countries and cultural contexts. Moreover, the participants might have thought of a specific airport that they are familiar with (e.g., Zurich airport), which might have influenced their responses. However, comparing prior studies in different countries did not suggest substantial differences in the variables of interest (Scurich and John 2014; Stotz et al. 2020). Third, the way randomised security checks are perceived might impact our results. From a basic psychological standpoint, there is a long-existing debate about human’s abilities to detect and recognise randomness (Ayton et al. 1989; Bar-Hillel and Wagenaar 1991; Hogarth 1987). People might not know how true randomness can be achieved and what that means for security, as was shown in Tamara Stotz et al. (2021) and Scurich and John (2014). In our study, we provided the participants with a clear instruction (that every third passenger is being checked) so that they have been able to form a mental image of how randomisation in this case was achieved. While we cannot guarantee that people understood this concept, it will likely be easier to picture, compared to simply informing them that a security check is randomised. More importantly, the participants might not have believed that the security checks were truly randomised and might have thought of other criteria to choose people to be checked (e.g., race). This would introduce other relevant factors, such as perceived fairness and ethical considerations. This would imply that the participants in our study considered their own chances of being chosen for the security check, instead of perceiving the check as truly randomised. This might have led to an overestimation of the likelihood of not being chosen for the security screening for some participants and underestimation for some participants (based on their assumptions of likelihood to being checked). Fourth the scale measuring the willingness to breach social norms did not exhibit a high internal reliability. Future studies should attempt to improve the scale and also uncover other individual factors that impact airport security perception and deterrence.

### Conclusions

In this study, we were interested whether the active communication of additional unpredictable security measures influences deterrence in general. Specifically, we were interested in the question whether the information of additional security measures decreases the detrimental effect of randomised security checks. Overall, the results support prior findings that suggest that people utilise heuristics, such as their affect triggered by certain cues and their expectations, when making decisions about security measures at airports (Finucane et al. 2000; Nguyen and John 2018; Pita et al. 2009).

## Data Availability

The data that support the findings of this study are available from the corresponding author upon request.

## Footnotes

1 The term ‘traditional’ is used for the type of security check that is currently implemented in most airports worldwide, where all passengers are screened. It is seen by most passengers as the status quo.
